# Analytical validation of a 12-gene molecular test for the prediction of distant recurrence in breast cancer

**DOI:** 10.4155/fsoa-2017-0051

**Published:** 2017-06-05

**Authors:** M Bryan Warf, Saradha Rajamani, Kristin Krappmann, Jennifer Doedt, Jared Cassiano, Krystal Brown, Julia E Reid, Ralf Kronenwett, Benjamin B Roa

**Affiliations:** 1 Myriad Genetic Laboratories, Inc., Salt Lake City, UT 84108, USA; 2 Myriad Genetics, Inc., Salt Lake City, UT 84108, USA; 3 Sividon Diagnostics GmbH, Cologne, Germany

**Keywords:** analytical validation, ER+/HER- invasive breast cancer, gene expression

## Abstract

**Aim:**

To validate the analytical performance of a 12-gene molecular assay that predicts distant recurrence for early-stage ER+/HER2- invasive breast cancer as run within a central reference laboratory.

**Materials & methods:**

Formalin-fixed paraffin-embedded breast resections were evaluated by quantitative reverse transcription polymerase chain reaction for the expression of eight target genes, three housekeeper genes and one control gene to assess for DNA contamination.

**Results:**

The assay results were highly correlated with a validated reference laboratory. The assay had a broad linear range for input RNA, with similar amplicon efficiencies for target and housekeeper genes. The assay test was highly reproducible, with comparable inter- and intrabatch precision to the reference laboratory.

**Conclusion:**

These studies demonstrate that the 12-gene molecular assay is highly robust and accurate.

## Lay abstract

Several tests have been developed to aid in breast cancer treatment decisions by assessing the likelihood that an individual’s breast cancer will recur. One such test is a 12-gene molecular assay. This test provides a clinical score that combines molecular information from genes within the tumor with clinical information to determine whether a breast cancer is at high or low risk of recurrence. The data presented here show that this test is very reproducible and robust, making it appropriate and reliable for clinical use.

Breast cancer is the most prevalent form of cancer among women, with over 240,000 cases of invasive breast cancer diagnosed in the USA in 2016 [1]. With many local and systemic treatment options available, treatment decisions have historically been based on clinical features, such as tumor pathology, nodal status and patient age [2]. Risk of distant recurrence (metastatic disease) is often a significant factor in determining whether systemic treatment should be sought after surgery, as the toxicity and comorbidity associated with chemotherapy may outweigh the potential benefit [2]. Unfortunately, clinical factors used to predict disease recurrence, including tumor grade, nodal status and Ki67 expression, often do not provide enough information to predict the efficacy of chemotherapy [3]. As there are a significant number of low risk breast cancers that will not exhibit oncologic progression [4], an estimated 20–40% of patients receive unnecessary systemic treatment.

Several molecular tests have been developed to improve prediction of breast cancer recurrence and aid in treatment decisions [5]. One such test is a 12-gene molecular assay (EndoPredict) that has been developed and validated for use in ER+/HER2- invasive breast cancer [6–10]. This test uses reverse transcription followed by quantitative polymerase chain reaction (RT-qPCR) to measure the expression of eight target genes, three housekeeper genes for normalization and one control gene to assess for DNA contamination. The differential gene expression of the target and normalization genes is used to calculate a molecular score, which is then combined with clinical parameters to determine a clinical score and an overall 10-year risk estimate of distant recurrence [6–9]. This clinical score has been shown to predict the risk of distant metastasis independent of clinical–pathological parameters, such as Ki67 and quantitative ER immunohistochemistry [8].

Rigorous analytical validation of RNA expression assays is required for clinical use. The 12-gene molecular assay was initially developed as a kit to be used by pathology laboratories across Europe. Previous studies of the analytical performance of this gene expression assay have involved a kit format run on the Versant kPCR platform [11–14]. Recently, a laboratory developed test (LDT) version of the expression signature that utilizes a different RT-qPCR platform (QuantStudio DX) was introduced for centralized testing in the USA. Here we present the analytical validation of the LDT. This includes the analytical precision and accuracy of the LDT as well as the dynamic range of the assay and stability of the input RNA.

## Materials & methods

### Tissue processing, RNA extraction & gene expression measurement

Samples were analyzed within a Clinical Laboratory Improvement Amendments certified laboratory (Myriad Genetic Laboratories, Inc., UT, USA). Archival formalin-fixed paraffin-embedded (FFPE) breast resection tissue of treatment-naive, invasive ER+/HER2- female breast cancer (Sividon Diagnostics GmbH, Cologne, Germany) was tested in these studies. All samples were anonymized prior to testing. Each case required one hematoxylin and eosin stained slide and at least one 10 μm section of unstained tissue.

During testing, the hematoxylin and eosin slide for each case was first reviewed by an anatomic pathologist to verify that the sample was suitable for testing and to identify the area of the lesion to be tested. The corresponding area of unstained tissue was then macrodissected and combined into a single tube. RNA was manually extracted using the magnetic bead-based Versant Tissue Preparation Kit (Siemens, Munich, Germany) following the manufacturer’s recommended protocol, which includes DNase treatment of the extracted RNA.

To measure gene expression of the eight target and three normalization genes, 45 μL of RNA was used to inoculate a 96-well plate (Sividon Diagnostics) that contained forward/reverse primers and carboxyfluorescein-labeled probes dried into the wells (Thermo Fisher Scientific, MA, USA). Gene expression measurements were made for the eight target genes of interest (*AZGP1*, *BIRC5*, *DHCR7*, *IL6ST*, *MGP*, *RBBP8*, *STC2* and *UBE2C*) and three housekeeper genes used for normalization (*CALM2*, *OAZ1* and *RPL37A*). An additional control gene (*HBB*) was used to detect contamination by residual DNA, which is sensitive only to genomic DNA. One-step RT-PCR was then performed using TaqPath enzyme on a QuantStudio DX instrument, according to the manufacturer’s specifications (Thermo Fisher Scientific).

All samples were run in triplicate and the expression values from each target and housekeeper gene were averaged. The average value for each target gene was then normalized by the average expression value of all three housekeeper genes (housekeeper mean [HKM]). A molecular score was then calculated as the linear combination of the normalized target gene expression [8]. Expression of the target and housekeeper genes was recorded as the crossing threshold (C_T_) value. A clinical score was then calculated by combining the molecular score with tumor size and the number of positive lymph nodes as previously described [8]. Samples with a clinical score ≤3.3 were considered low risk for distant recurrence, while samples with scores ≥3.4 were considered high risk for recurrence [8].

The data quality for each sample and control were analyzed, and only samples (with controls tested alongside the sample) with good quality data generated a passing molecular score [8]. The average expression value of the three housekeeper normalization genes (HKM) was used to determine if the sample was within the validated range of the test (HKM values of 19–27). Samples with an HKM outside of this range would be failed. Samples with two or more replicates that generated a C_T_ for *HBB* below 38 would be considered contaminated by genomic DNA and would require retesting after additional DNase treatment; however, no samples in these validation studies required additional DNase treatment.

#### Laboratory control processes

Specific control processes are implemented within our Clinical Laboratory Improvement Amendments-certified laboratory to ensure reproducibility and accuracy. These control processes are codified within standard operating procedures that are maintained through a change in control quality system. In brief, every batch of one or two clinical samples is tested with a positive control RNA, a negative nontemplate control and an internal control for DNA contamination. These controls have known performance metrics used to qualify clinical samples. Incoming reagents are tested against previously qualified reagents and stored until use (in one-use aliquots). New or serviced instruments are qualified prior to use, according to installation and operational qualification standard operating procedures. Proficiency testing for technicians is performed on an annual basis and for the assay on a biannual basis.

### Accuracy of LDT platform

Forty FFPE breast resection samples were tested by the LDT laboratory (Myriad Genetic Laboratories) and the reference laboratory (Sividon Diagnostics). Reference laboratory testing methods have been previously described [8,11]. Results were blinded until gene expression testing was complete. Molecular scores determined by the LDT and reference laboratories were compared for each sample. Differences in the molecular and clinical scores calculated for each sample by the LDT laboratory and the reference laboratory were tested using paired t-test and Lin’s concordance correlation test. All analyses were performed with a two-sided significance level α = 0.05. All statistical analyses were performed using R, version 3.3.0.

### Precision estimation

The inter- and intrabatch precision of the clinical score was assessed in archival samples (Sividon Diagnostics) with sufficient tissue to produce replicate samples for testing. Interbatch precision was assessed by testing a set of 12 samples, with three replicates for each sample. Each sample replicate was tested from new tissue and processed on a different day with a new 96-well plate. The intra-batch precision was assessed in a set of 20 samples, with the same RNA from each sample tested twice on the same 96-well plate. The standard deviation (SD) was determined for the inter- and intrabatch precision by calculating the square root of the observed mean variance of the samples within the dataset. A two-sided 95% confidence bound was calculated from the distribution.

### Assessment of RNA input linear range & complementary DNA amplification efficiency

The linear range for the RNA input was determined for the molecular score as well as for each target and normalization gene, using a positive control sample (aggregate RNA sample from homogenized normal human tissue, Agilent Technologies, CA, USA). The RNA concentration in the positive control sample was first quantitated using a Nanodrop spectrophotometer (Thermo Fisher Scientific). Serial dilutions were then prepared for the sample, with final RNA concentrations ranging from 100 to 0.049 ng/μL (RNA input amounts from 4500 to 2.2 ng), which were determined to correspond to an HKM of 17–28.

The linear range was defined as the range of RNA concentrations over which the R^2^ value for a linear fit model (plotting C_T_ vs the log concentration) was ≥0.95 for all eight target and three normalization genes. Using the positive control sample, amplification efficiencies for each gene were also determined by using the formula:(1)
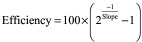



wherein the slope is estimated from the regression of C_T_ values versus log base 2 of the RNA concentration, over the previously determined linear range.

### Stability of extracted RNA

RNA was extracted from six archival samples (Sividon Diagnostics) and aliquoted. One aliquot for each sample was tested initially at time zero. The remaining aliquots were stored at -20°C and tested over a 6-week period. The SD of each sample was determined as the square root of the observed mean variance of all the timepoints, with a two-sided 95% CI

## Results

### Accuracy of the LDT

**Figure 1.**
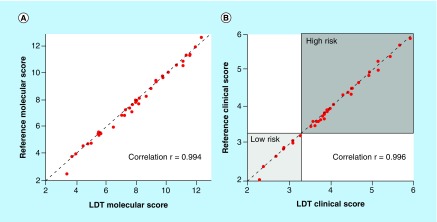
**The molecular and clinical scores are concordant for samples tested by both laboratory developed test and reference laboratories.** **(A)** Molecular scores and **(B)** clinical scores are graphed for paired samples that were tested by both the LDT and reference laboratories. For the clinical scores, scores that are ≥3.4 and are within the dark gray box are high risk, while scores that are ≤3.3 and are within the light gray box are low risk. All samples had concordant risk classifications when comparing the LDT and reference laboratory. LDT: Laboratory developed test.

The accuracy of the molecular and clinical scores produced by the LDT was evaluated by testing 40 samples that were previously tested by the reference laboratory and had passing scores. Out of the 40 tested samples, 37 produced passing scores within the LDT laboratory. The molecular scores between the two laboratories were highly correlated, with a Lin correlation coefficient of 0.994 (95% CI: 0.988–0.997; Figure 1A). Similarly, the correlation coefficient for the final clinical scores was 0.996 (95% CI: 0.992–0.998; Figure 1B).

The risk categorization for distant recurrence (low vs high risk) was concordant between the two laboratories for all 37 samples (Figure 1B). The mean difference in scores between the two laboratories was determined to be 0.16 score units (95% CI: 0.084–0.24) for the molecular score and 0.045 score units (95% CI: 0.023–0.068) for the clinical score. These differences are similar to the SD of the test for both the reference laboratory [11] and the LDT laboratory (see the 'Precision' section).

### Precision

The interbatch precision of the LDT was estimated using a set of 12 samples, with three biological replicates for each sample tested on a different day with new tissue. All 36 replicates for these 12 samples produced passing results. The interbatch SD was determined to be 0.21 score units (95% CI: 0.15–0.35) for the molecular score and 0.057 score units (95% CI: 0.041–0.098) for the clinical score (Supplementary Table 1).

The intrabatch precision was estimated in a set of 20 samples, with the RNA from each sample tested twice on the same 96-well plate. We determined that the intrabatch SD was 0.079 score units (95% CI: 0.056–0.13) for the molecular score and 0.022 score units (95% CI: 0.016–0.038) for the clinical score (Supplementary Table 2). These results are similar to the previously validated kit format [11] and demonstrate that the LDT is similarly reproducible.

The precision of each individual gene was determined, using the interbatch precision dataset. We observed that all genes were similarly precise, with a median SD of 0.31 C_T_ units for the three housekeeper genes and 0.33 C_T_ units for the eight target genes (Table 1). These values for the LDT cannot be directly compared with the kit, as this gene-specific analysis was not performed when assessing the analytical validity of the kit format of this test [11].

**Table 1.** **Performance characteristics for target and housekeeper genes.**

**Gene**	**Amplicon efficiency % (95% CI)**	**Linearity R^2^**	**SD (95% CI) (C_T_)**
**Target genes**			
*AZGP1*	97.6 (89.3–107.5)	0.982	0.31 (0.22–0.53)
*BIRC5*	98.8 (92.3–106.2)	0.989	0.35 (0.25–0.59)
*DHCR7*	99.3 (93.0–106.5)	0.990	0.32 (0.23–0.56)
*IL6ST*	98.1 (92.9–103.9)	0.993	0.33 (0.23–0.56)
*MGP*	97.0 (91.0–103.9)	0.990	0.36 (0.25–0.61)
*RBBP8*	98.9 (90.6–108.8)	0.983	0.33 (0.24–0.56)
*STC2*	101.1 (94.1–109.1)	0.988	0.33 (0.24–0.57)
*UBE2C*	95.9 (90.7–101.7)	0.993	0.31 (0.22–0.53)
Median	98.5	0.990	0.33
**Housekeeper genes**			
*CALM2*	101.0 (96.2–106.2)	0.995	0.33 (0.24–0.57)
*OAZ1*	101.1 (96.6–106.1)	0.995	0.31 (0.31–0.52)
*RPL37A*	97.8 (91.5–105.1)	0.990	0.29 (0.21–0.50)
Median	101.0	0.995	0.31

### Linearity

It is important to define the acceptable range of input RNA concentrations over which the genes within the signature yield accurate and consistent results. During clinical testing, the input RNA is assessed by measuring the averaged expression of the three housekeeper normalization genes (HKM). This gene expression signature has been validated for samples with HKMs from 19 to 27.

In order to ensure that the LDT is accurate over the validated HKM range, the RNA input linearity was assessed by testing a dilution series of the positive control RNA. We first determined that the validated HKM range of 19–27 correlated approximately to RNA concentrations of 18.5–0.16 ng/μL (RNA input range of 832.5–7.2 ng) for the positive control RNA. We then performed a dilution series and tested the positive control over a broader range of concentrations, from 100 to 0.049 ng/μL of RNA (corresponding to an HKM range of ∼17–28).

**Figure 2.**
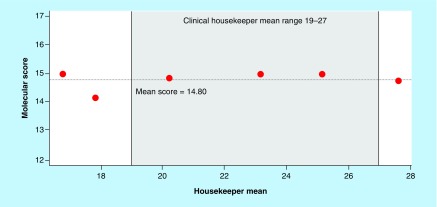
**RNA input linearity.** The molecular scores for the dilution series of the positive control are plotted against the HKM measured for each dilution point. The HKM range over which the assay was previously validated (19–27) is shaded in gray. The mean score for all dilution points is listed and indicated by a dotted line across the graph. HKM: Housekeeper mean.

We calculated the linear range of the LDT by determining the range over which each of the target and normalization genes was linear, as defined by having an R^2^ value ≥0.95 when plotting C_T_ versus concentration. We found that each gene had R^2^ values >0.98 over the input RNA concentration range (100–0.049 ng/μL; Table 1, Supplementary Figure 1) with a maximum deviation from a linear fit model of only 0.85 C_T_s. The molecular score was also consistent over this range (Figure 2), with an SD of 0.34 (95% CI: 0.21–0.84). These data indicate that the linear RNA concentration range for the LDT is at least from 100 to 0.049 ng/μL (HKM range of ∼17–29), which is broader than the validated range over which clinical samples are reported (HKM range of 19–27).

### Amplification efficiency

The amplification efficiency of each of the target and normalization genes within the signature was also determined over the linear range (HKM range of ∼17–29). Amplification efficiencies ranged from 95.9 to 101.1%, with median efficiencies of 98.5% for the target genes and 101.0% for the housekeeper genes (Table 1). We observed no statistical difference in the amplification efficiencies when comparing housekeeper and target genes (p = 0.26). These values are similar to the previously reported amplification efficiency values for kit format [11].

### Stability

**Figure 3.**
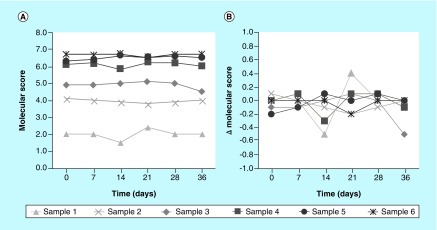
**Stability of extracted RNA.** **(A)** Molecular score and **(B)** Δ molecular score are shown for the time course, for each of the six tested sample. The Δ score represented the difference between the score at that time point and the average score for that sample over the 6-week time period.

During the course of clinical testing, extracted RNA samples are destroyed one month after extraction. Samples that require re-testing after a month must utilize RNA extracted from new tissue. The stability of the extracted RNA was tested to ensure that scores were reproducible over at least the storage time frame. To this end, RNA was extracted from six samples and tested over a 6-week period (Figure 3). These six samples had a range of molecular scores, from approximately 2 to 7. We observed that the molecular scores were reproducible across all time points and that all samples had an SD ≤0.28 score units over the 6-week time period. This was similar to the overall SD measured for the LDT (see the 'Precision' section).

## Discussion

Given the limited benefit of chemotherapy in women with a low risk of breast cancer recurrence, there is a need for adjuvant testing to aid in treatment decisions. Although such treatment decisions have historically utilized only clinicopathologic information, it is well known that these features are insufficient to accurately plan treatment for many patients [3]. In response, a 12-gene molecular assay was developed and validated to differentiate patients with a high and low risk of distant breast cancer recurrence [8]. It is critical that the analytical performance of gene expression assays be proven in order to ensure robust and accurate test results. The analytical performance of the 12-gene molecular assay has been previously validated for the qRT-PCR kit platform utilized in Europe. Here we assessed the analytical performance of a recently developed LDT, which utilizes the QuantStudio DX platform for RT-qPCR.

We observed that the LDT was highly accurate, with highly correlated molecular and clinical scores relative to the validated reference laboratory. Although there was a statistically significant difference between scores determined at the LDT and reference laboratories, this significance was likely due to the high power of the study. The 0.16 difference in molecular scores observed between the reference and LDT laboratories is similar to the overall SD measured for the test, as run in either laboratory.

The LDT was determined to be very reproducible, with an SD of 0.16 score units for the molecular scores and only 0.045 score units for the clinical score, representing about 0.3% of the clinical reporting range. Overall, these data show that the molecular and clinical scores determined by the LDT laboratory are highly accurate and reproducible.

The reference laboratory has previously validated the 12-gene molecular assay for a range of HKMs between 19 and 27. Here we observed that the LDT assay was reproducible over an HKM range of at least 17–28, with R^2^ values >0.98 for all target and housekeeper genes over this range. This corresponds to over a 2000-fold dilution range, with input RNA concentrations ranging from 0.049 to 100 ng/μL. Overall, this demonstrates that the LDT has a dynamic range much broader than the clinical testing range.

Laboratory samples are re-tested when control or technical failures occur. As such, RNA samples are stored for a 1-month time period by the laboratory. It is important to ensure that accurate scores are generated for samples re-tested over this time period. We observed that RNA samples extracted from FFPE tissue have reproducible molecular scores over at least a 6-week window, with an SD of only 0.28 score units. This indicates that the LDT reproduces consistent scores for samples stored over a longer time frame than that over which clinical samples are re-tested.

## Conclusion

Overall, these studies demonstrate that the 12-gene molecular assay run on the QuantStudio DX platform is a reproducible and robust test. Both the dynamic range and sample stability surpass clinical testing parameters. Combined with the previous clinical validation studies, this analytical validation demonstrates that the LDT version of the 12-gene molecular assay can be utilized for evaluating risk of distant recurrence in ER+/HER2- invasive breast cancer.

## Future perspective

It is becoming increasingly clear that a personalized approach to the management of cancer can drastically improve patient outcomes. A variety of different tests need to be developed to help determine which specific treatments will be beneficial to each unique patient for personalized medicine to be successful across the broad field of oncology. To this end, complex molecular testing of FFPE lesions has proven to be highly effective in identifying which patients with ER+/HER2- breast cancer should receive additional chemotherapy. It is likely that these types of molecular tests will become the backbone of personalized treatment of all cancers. However, it is imperative that each test has both extensive clinical and analytical validations, to ensure that patients receive appropriate and effective treatments.

**Summary points**

- This work represents the analytical validation of a molecular assay laboratory developed test (LDT) that uses RNA expression of eight target genes to identify patients with early-stage ER+/HER2- breast cancer who are at low risk of disease recurrence and may not benefit from chemotherapy.
- The molecular and clinical scores produced by the LDT were highly correlated with scores generated by the validated reference laboratory, with Lin correlation coefficients >0.99.
- The interbatch SD was 0.21 score units for the molecular score and 0.057 score units for the clinical score. Similarly, the intrabatch SD was 0.079 and 0.022, respectively.
- The molecular score was reproducible over at least a 2000-fold range of RNA concentrations for housekeeper means between 17 and 28, which is wider than the range over which clinical samples are reported.
- The median amplification efficiency was 98.5% for target genes and 101.0% for normalization genes.
- Extracted RNA is stable under current storage conditions at -20°C, generating reproducible molecular scores over at least a 6-week time frame.
- Overall, these studies show that the 12-gene molecular assay LDT is robust and reproducible.
