# Supplementary material for: Analytical validation of a 12-gene molecular test for the prediction of distant recurrence in breast cancer
Source: Future Sci OA. 2017 Jun 5;3(3):FSO221. doi: 10.4155/fsoa-2017-0051 (PMC5583651; doi:10.4155/fsoa-2017-0051)
Supplement: Supplementary file 1 [file fsoa-03-221-s1.docx]

**Supplemental Table 1. Inter-Batch Precision.** The molecular and clinical scores are shown for the three replicates of each of the 12 samples used to determine inter-batch precision.

|  | **Molecular Score** | | | **Clinical Score** | | |
| --- | --- | --- | --- | --- | --- | --- |
| **Sample No.** | **Replicate 1** | **Replicate 2** | **Replicate 3** | **Replicate 1** | **Replicate 2** | **Replicate 3** |
| 1 | 12.7 | 12.4 | 12.7 | 5.3 | 5.2 | 5.3 |
| 2 | 6.5 | 6.2 | 6.3 | 4.5 | 4.4 | 4.5 |
| 3 | 4.5 | 4.7 | 4.3 | 3.6 | 3.6 | 3.5 |
| 4 | 8.9 | 9.0 | 8.7 | 3.8 | 3.9 | 3.8 |
| 5 | 4.1 | 4.0 | 4.0 | 3.1 | 3.1 | 3.1 |
| 6 | 10.9 | 11.2 | 10.8 | 5.1 | 5.2 | 5.1 |
| 7 | 8.7 | 8.3 | 8.4 | 3.8 | 3.7 | 3.7 |
| 8 | 11.6 | 11.4 | 11.4 | 4.6 | 4.5 | 4.5 |
| 9 | 11.6 | 11.2 | 11.5 | 5.9 | 5.8 | 5.9 |
| 10 | 7.2 | 6.7 | 7.3 | 3.7 | 3.6 | 3.7 |
| 11 | 2.9 | 2.8 | 3.4 | 2.2 | 2.1 | 2.3 |
| 12 | 10.9 | 11.0 | 10.7 | 4.7 | 4.8 | 4.7 |

**Supplemental Table 2. Intra-Batch Precision.** The molecular and clinical scores are shown for the two replicates of each of the 20 samples used to determine inter-batch precision.

|  | **Molecular Score** | | **Clinical Score** | |
| --- | --- | --- | --- | --- |
| **Sample No.** | **Replicate 1** | **Replicate 2** | **Replicate 2** | **Replicate 3** |
| 1 | 9.8 | 9.7 | 4.1 | 4.1 |
| 2 | 8.1 | 8.3 | 4.0 | 4.0 |
| 3 | 9.8 | 9.9 | 4.4 | 4.5 |
| 4 | 5.6 | 5.4 | 3.5 | 3.5 |
| 5 | 4.9 | 4.8 | 2.7 | 2.7 |
| 6 | 5.6 | 5.7 | 2.9 | 2.9 |
| 7 | 9.4 | 9.4 | 5.0 | 5.0 |
| 8 | 8.3 | 8.2 | 3.7 | 3.6 |
| 9 | 7.1 | 7.1 | 3.7 | 3.7 |
| 10 | 9.4 | 9.4 | 4.3 | 4.3 |
| 11 | 5.4 | 5.1 | 3.2 | 3.1 |
| 12 | 8.0 | 8.1 | 3.9 | 4.0 |
| 13 | 7.6 | 7.7 | 3.8 | 3.9 |
| 14 | 7.8 | 7.9 | 3.9 | 3.9 |
| 15 | 3.8 | 3.7 | 2.4 | 2.4 |
| 16 | 5.7 | 5.7 | 3.3 | 3.3 |
| 17 | 8.0 | 8.0 | 3.9 | 3.9 |
| 18 | 8.2 | 8.2 | 4.0 | 4.0 |
| 19 | 11.7 | 11.6 | 5.0 | 4.9 |
| 20 | 10.8 | 10.9 | 4.7 | 4.8 |

**Supplemental Figure 1. RNA input linearity.** The C_T_ value for the dilution series of the positive control are for each target and housekeeper gene. The R^2^ value for a linear fit model for is listed for each gene. The associated HKM is listed for each dilution point.

**
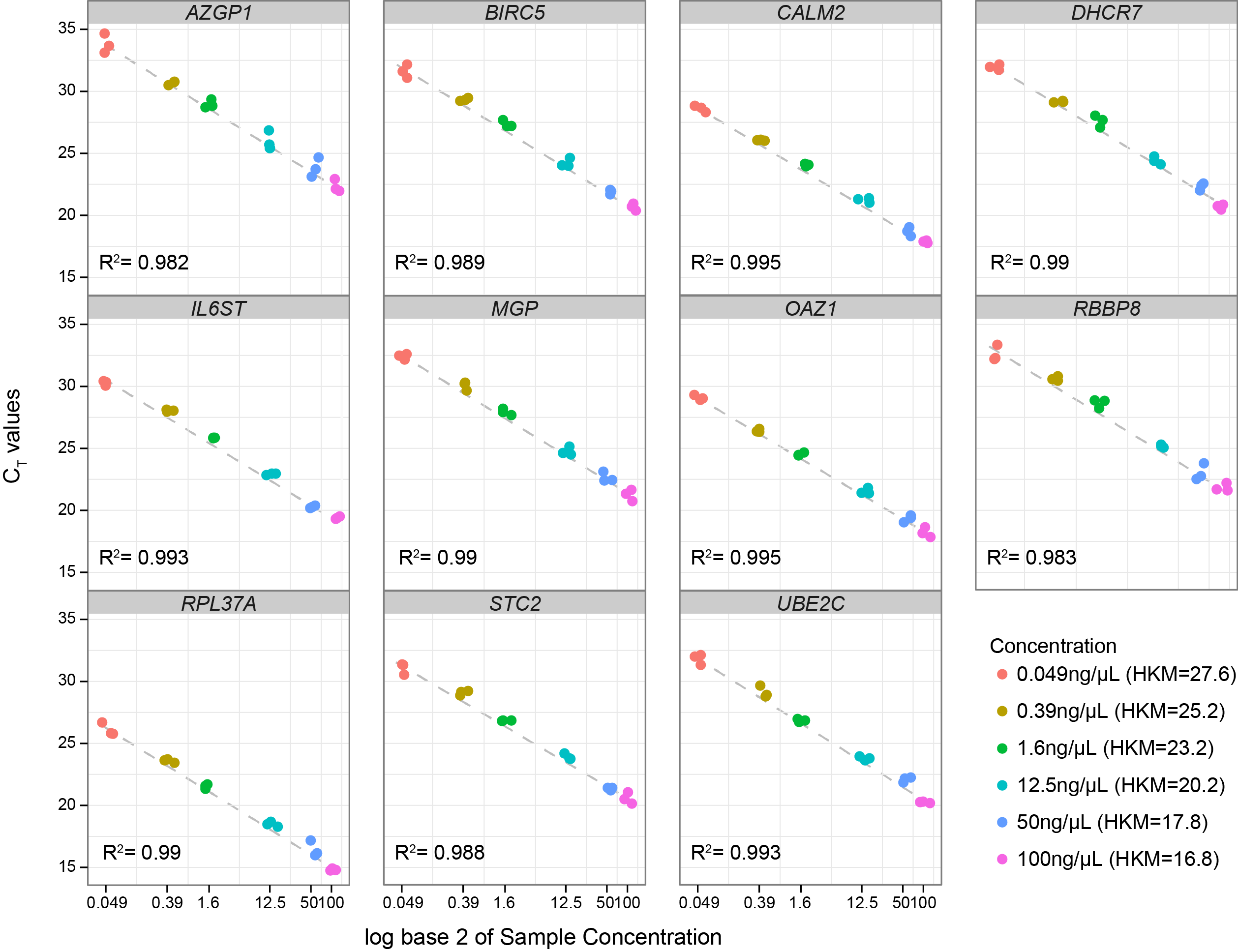
**
